# Supplementary material for: Confirmation of the southern African distribution of the marine sponge Hymeniacidon perlevis (Montagu, 1814) in the context of its global dispersal
Source: PeerJ. 2022 Nov 25;10:e14388. doi: 10.7717/peerj.14388 (PMC9703993; doi:10.7717/peerj.14388)
Supplement: Appendix S1 [file peerj-10-14388-s001.pdf]

Global pattern in the distribution of exotic sponge *Hymeniacidon perlevis*

Dawit Yemane and Toufiek Samaai

2022-06-01 14:32:59

Contents

1 Summary **2**

2 Introduction **2**

3 Methods **2**

3.1 Statistical models . . . 3

3.1.1 Classification Tree Analysis (CTA) . . . 4

3.1.2 Generalized Linear Model (GLM) . . . 4

3.1.3 Generalized Additive Models (GAM) . . . 4

3.1.4 Generalized Boosted Model (GBM) . . . 4

3.1.5 Multivariate Adaptive Regression Spline (MARS) . . . 4

3.1.6 Random Forest (RF) . . . 5

3.1.7 Support Vector Machine (SVM) . . . 5

3.1.8 Artificial Neural Network (ANN) . . . 5

4 Results **5**

4.1 Explore: sponges occurrence . . . 5

4.2 Modelling sponge distribution . . . 6

5 Discussion **13**

6 Appenedix **13**

6.1 Appendix A . . . 13

6.2 Appendix B . . . 18

7 Session information **27**

References **28**

1 Summary

This report is intended to provide an overview of the global pattern of distribution of exotic sponge *Hymeniacidon perlevis.* Occurrence records from multiple sources, OBIS records, genbank, and local South African monitoring, were utilized to develop distribution model for this species. Global and readily available environmental layers were downloaded and processed to be used as predictors in modelling the distribution of *Hymeniacidon perlevis.* An ensemble modelling approach was adopted whereby multiple species distribution models were combined, weighted by their predictive performance, to create an ensemble distribution map. To model the following commonly used statistical model were used: Generalized Additive Models (GAM),Classification Tree Analysis (CTA),Generalized Linear Model (GLM),Generalized Boosted Model (GBM),Multivariate Adaptive Regression Spline (MARS),Random Forest (RF), Artificial Neural Network (ANN), and Support Vector Machine (SVM).Mean surface temperature was the most important predictor of the distribution of *Hymeniacidon perlevis* globally, followed by the range of surface temperature. Most of the models performed reasonably well with AUC mostly above 0.8.

2 Introduction

Sponges are important of marine ecosystem both providing habitat for other marine organisms, important component of the deep sea ecosystem, and bioprospecting for pharmaceutical uses. *Hymeniacidon perlevis*, despite its limited dispersal capability, is considered common and widely distributed (Turner, 2020).

Previous work on the distribution of *Hymeniacidon perlevis* - regional and/global studies.

[Comprehensive review of previous work on the distribution of sponges in general and environmental and ecological processes regulating their distribution]. Although *Hymeniacidon perlevis* common and widely distributed species of sponge, evidence of which is largely drawn from genetic work, there has not really been large scale species distribution modelling based on their recorded occurrences. This study attempts to fill this gap to an extent. Due to the paucity of occurrence data on *Hymeniacidon perlevis* this study relies on occurrence data from multiple sources, OBIS, genbank, and South African coastal monitoring work, to develop the distribution model.

[background on the study - ADD] [study objective - ADD]

3 Methods

The study region, sampling method, data processing and preparation for modelling distribution of the sponge species are given below.

[Description of study area/region]

Map of the study region with sampling with locations of occurrence record. Description of the sampling method, gear and processing of samples.

[Description of data processing]

Processing of the raw occurrence data for modelling distribution of sponges.

[Preparation and processing of environmental data]

[Preparation of occurrence data]

Raw occurrence/encounter, from the three different sources mentioned above, were used in modelling the distribution of *Hymeniacidon perlevis.* As noted below the analysis conducted with and without spatial thinning (to reduce spatial bias due to non random sampling and reduce spatial autocorrelation).

[Generation of background data/pseudo-absence]

The species distribution modelling approach we followed mostly relies on *SSDM* (**R-SSDM?**) R package. The *SSDM* package allows for: modeling distribution of species using one of the nine correlative statistical models, and combining them into an ensemble species distribution map. Prediction from individual models were combined into an ensemble species distribution maps by first filtering out model with predictive performance below set threshold and then combining the remaining as weighted mean (individual model performance taken as a weight). As measure of model performance, e.g the Area Under the Curve (AUC) a widely used measure of performance for classification models, True Skills Statistic (TSS), Kappa, and others will be used to weigh individual models when creating ensemble distribution map.

It is uncommon to find spatial auto-correlation of varying magnitude (and hence varying level of problem unless accounted for in the model) in the residuals from species distribution model. There a range of approaches to either explicitly model spatial autocorrelation or account for it. To reduce the effect of spatial bias, while still keeping most of the information in the data, *SSDM* allows for spatial thining. The selection of the numbers of pseudo-absences were based on recommendation of (Barbet-Massin *et al.*, 2012) which was adopted in the *SSDM* package (**R-SSDM?**).

[Preparation of cross-validation data, model fitting and evaluation]

The standard holdout-training cross-validation was applied for assessing model performance/evaluation. Each of the models were fitted 4 times (for computatitonal reason limited). All standard model evaluation metrics were computed including: Area Under the Curve AUC, *Kappa*, TSS ,*Sensitivity*, and *Specificity*. But as noted above only the AUC of a model was used for selecting into and generation of the ensemble distribution map.

[Relative importance of variables]

Variable importance were computed on the holdout set. Importance of a variable was measured by how much correlation changes between predicted values before and after permuting (reshuffling) the variable in question (expressed in percentage).

$$I_v = 1 - Cor(P_f, Pv)$$

where I_v is index of importance of a variable, Cor is correlation coefficient, P_f is prediction from the full model, P_v is prediction after permuting/reshuffling the variable v.

Partial effect of each of the predictor were computed by predicting the response variable for the variable of interest while holding the other predictors at their mean.

[Ensemble forecasting]

Ensemble forecasting/modelling was constructed, as indicated above, as a weighted mean distribution map with the AUC of the models used as a weight. Only models with $AUC > 0.7$ were included when building the ensemble.

3.1 Statistical models

Although most of the statistical models used in this study are widely known and used in marine and fisheries ecology brief summary of the statistical algorithms is given below. There are large number of publications on species distribution modelling, using some or all of the statistical models described below, to note few: Elith et al. (2007), Leathwick et al. (2006), Shabani et al. (2016), and Barbet-Massin *et al.* (2012). A detailed review species distribution modelling, including on data processing, methods for selecting pseudo-absences/background occurrence location, model evaluation, generation of ensemble maps, can be found in multiple publications [Refs]. In the context selection of size of occurrence data, Norberg et al. (2019) found substantial difference in the performance of species distribution models especially for community where large number of species are rare.

3.1.1 Classification Tree Analysis (CTA)

Classification Tree Analysis (CTA), contrary to what Regression Tree Analysis does, is used to model qualitative response variables (in the context of this study to model presence/absence of modeled species). Recursive binary classification are used to grow trees, with classification error rate used as splitting criterion. The algorithm aim to assign observations in a region to the most commonly occurring class in that region, in the training set. This makes the classification error will be fraction of the observations in that region that do not belong to the most common class in the training set. Due to the limitation of the mis-classification error as a measure of quality of splits two other measures are used: Gini-index and cross-entropy (James et al. 2013).

3.1.2 Generalized Linear Model (GLM)

GLM generally refer to wide class of statistical models including simple linear, multiple linear, and logistic regression as special case (James et al. 2013). GLMs allow for modelling response variables generated by distribution among others including gaussian, poisson, binomial, Gamma, quasipoisson, ... etc. GLMs are composed of three main structure: error structure (identifying the distribution that generated the data); and linear predictor (includes linear sum of the effects of one or more independent variables with information about the independent variables); link function (specifies the relationship between the linear predictor and the expected mean of the response variables) (Gerrard and Johsnson, 2015).

3.1.3 Generalized Additive Models (GAM)

Generalized Additive Models (GAMs) are generalization of GLMs as it allows for non-linear effect of each variables, by representing the linear effect with smooth function, while maintaining additivity (James et al. 2013). One limitation of GAM is the potential to miss interactive effects in situation when there many predictors, as GAMs are by design additive. Although when needed interactive effects can be included manually (James et al. 2013).

3.1.4 Generalized Boosted Model (GBM)

CTA tend to suffer from high variance, there are multiple approaches developed to improve on standard CTA this includes GBM. Boosting involves growing of multiple trees sequentially on the modified version of the original data. Where information from previously grown tree is used (actually subsequently fitting to the residual from the previous tree). Briefly the process of fitting GBM follows this steps: 1) Standard CTA is fitted to the training set first 2) residuals from this will be used as response in the second Tree model 3) fits from this will be combined with prior tree model 4) residuals from this combined model will be used as response for building the next tree model 5) this is then repeated N numbers of times (N - to be specified by the user). Fits from all the trees are then combined to obtain final prediction from the GBM model.

3.1.5 Multivariate Adaptive Regression Spline (MARS)

MARS represent an iterative specification of splines especially important in cases when there are many predictors. It uses pairs of piece-wise linear functions with single knots (Azzalini and Scarpa, 2012). It is widely used in various fields. MARS fits interaction between pairs of predictors automatically and selection of model complexity (location knots and basis function) is done as part of the model fitting process, selecting model with biggest reduction in residual sums of square. MARS also allows to model effect of predictor(s) on multiple response variables simultaneously (e.g. in the context of this study modelling the distribution of all species as function of all the environmental jointly) (Leathwick et al. 2006).

3.1.6 Random Forest (RF)

The basis of random forest is standard decision tree but random forest improves upon the predictive accuracy of standard decision trees by building a large numbers of decision trees (hundreds to few thousands) based on the training data, in addition to remove correlation among trees random samples of predictors are used when considering a split in a tree and only one of these random subset of predictors is used in a split. Compared to other statistical methods random forest models are not prone to the problem of multi-collinearity; implicitly allow for interactive effects; can handle non-linear effects (James et al. 2013).

3.1.7 Support Vector Machine (SVM)

SVMs were initially developed for classification purpose and has since been developed and used in a range of applications. SVM is an improvement over previous classifier, maximal marginal classifier, which requires classes be separable by linear boundary. SVM on the other hand can deal with non-linear class boundaries. Although their are initially intended for classification of two classes (presence and absences as in the context of this study). But it has now been extended to deal with multiple classes.

3.1.8 Artificial Neural Network (ANN)

ANN when it was initially developed, as rough computational representation of the human brain, and typically comprises three interconnected nodes of layers: input layer (containing one neuron per input variable), hidden (number vary and can be optimally selected), and output layer (in the case of binary output two nodes). Each neurons in the hidden layer receive information from the input layer and then sums it and transforms it using fixed function (typically sigmoid function). ANN can be used both in the context of regression and classification. ANN are by default non-linear models flexible enough to account any smooth function (Thuiller *et al.*, 2009).

All the analysis, visualization and report generation were done in R (R Core Team, 2022). Multiple R packages were utilized for data processing, visualization, analysis and summary of results including (Alathea, 2015; Allaire *et al.*, 2022; Henry and Wickham, 2020; Robinson *et al.*, 2022; Wickham *et al.*, 2022b, 2022a; Xie, 2022; **R-lubridate?**).

The following model was fitted to model occurrence of the sponges.

Model	formula
occurrence	$sponge_{occ} \sim T\_mean + T\_range + S\_mean + S\_range + V\_mean$

where $sponge_{occ}$ is the occurrence is of *Hymeniacidon perlevis*; T_{mean} and T_{range} are the mean and range of coastal surface temperature respectively; S_{mean} and S_{range} are the mean and range of coastal surface salinity respectively; V_{mean} is the mean coastal surface current velocity.

4 Results

4.1 Explore: sponges occurrence

Visual exploration of raw occurrence data for *Hymeniacidon perlevis* from the three sources, OBIS, genbank, and local South African coastal monitoring, are shown in Figure 1. As can be seen in Figure 1 most of the occurrence records come from coastal area around the UK and South Africa, with the rest of the remaining occurrence records coming different parts of the globe.

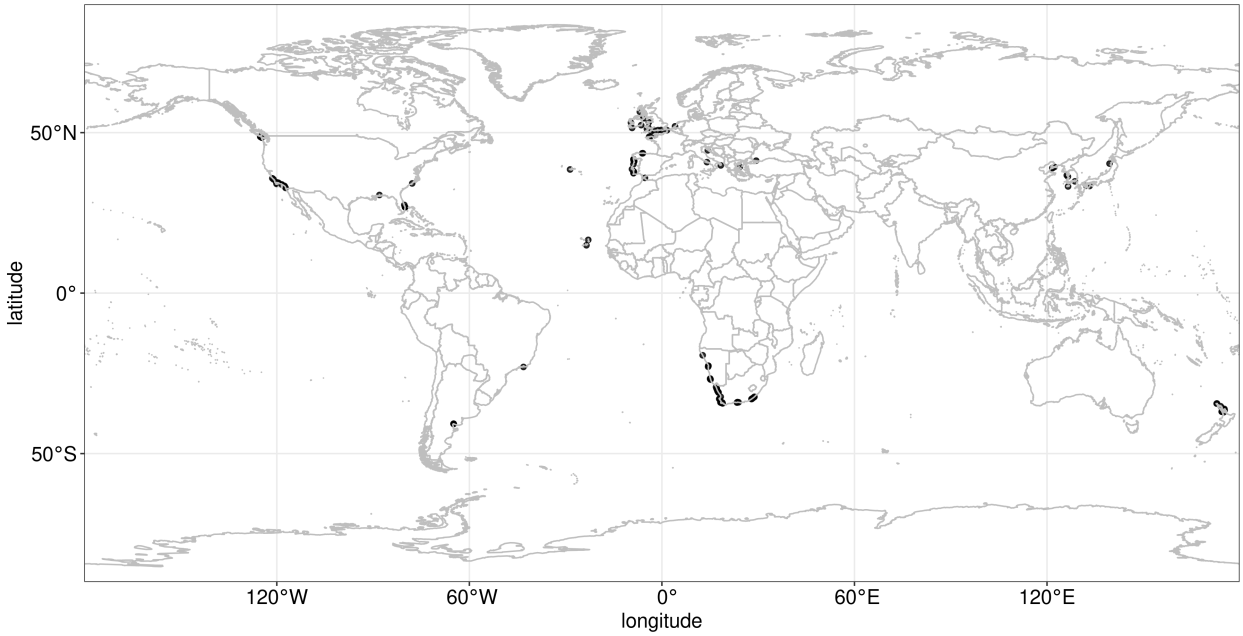

Figure 1: Map of the world with where *Hymeniacidon perlevis* are detected.

4.2 Modelling sponge distribution

Result from modelling the distribution of *Hymeniacidon perlevis* are presented below. Relative importance of predictors are presented in Figure 2 and Figure 3. Figure 2 presents overall importance of predictors from the ensemble results on the other hand model specific index of relative importance,combined with the ensemble, are presented in Figure 3.

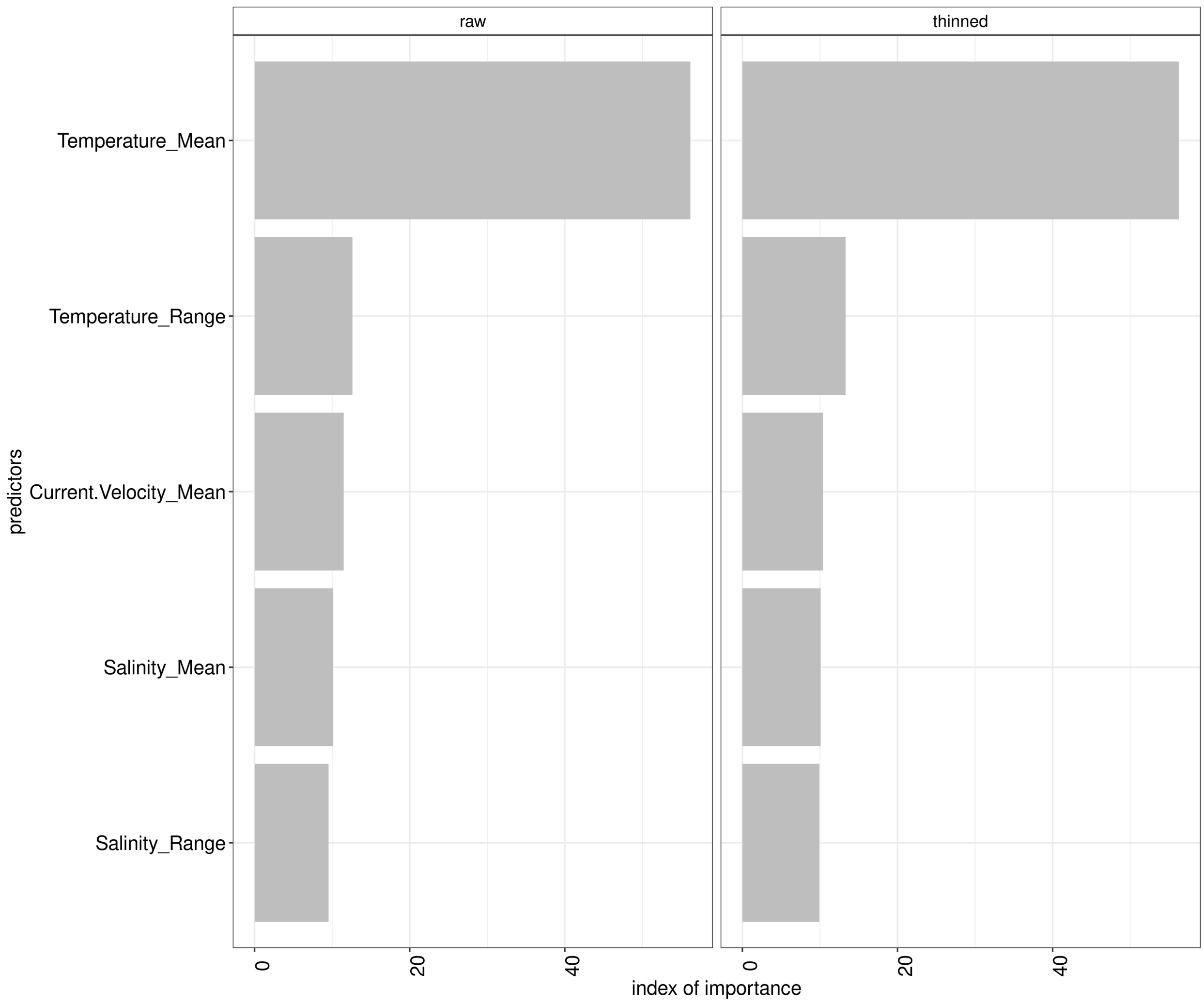

Figure 2: Relative importance of predictors in influencing the distribution of *Hymeniacidon perlevis* in the ensemble. Shown for the thinned and raw data

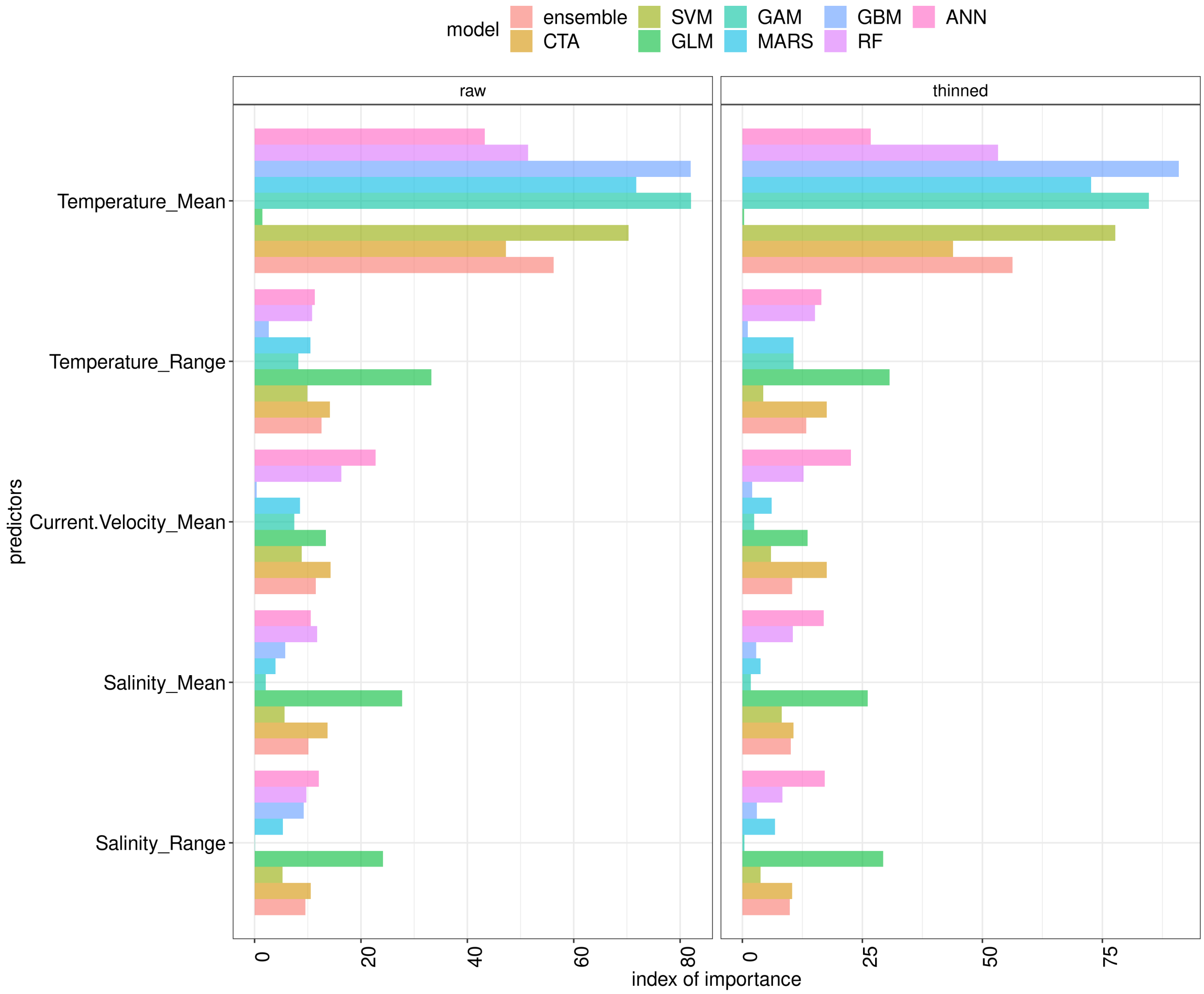

Figure 3: Relative importance of predictors in influencing the distribution of *Hymeniacidon perlevis* in each of the seven individual models and the ensemble. Shown for the thinned and raw data

Predictive performance of all the eight models considered and the ensemble models are shown in Figure 4. As can be seen in Figure 4 for the raw data random forest appear to perform better than the ensemble model whereas for the thinned data most models performed equally to each other and to the ensemble.

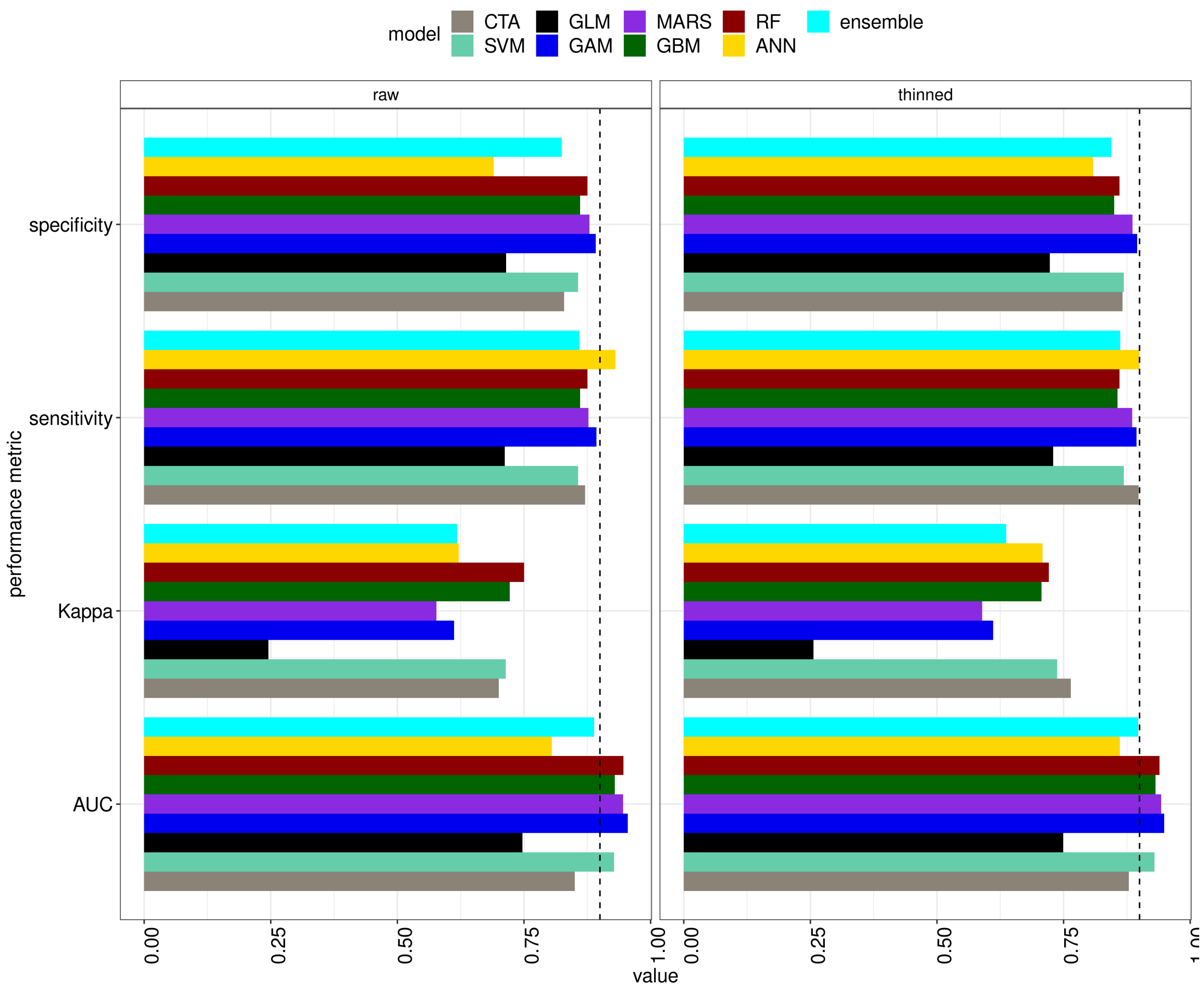

Figure 4: Predictive performance of each of the eight models, as measured by four measure of performance, and the ensemble. Shown for the thinned and raw data.

Partial effects of the five predictors on the probability of occurrences of *Hymeniacidon perlevis*, from the eight models are shown in Figure 5. The form of the Partial effects of mean and range of coastal surface temperature, the two most important variables affecting the probability of occurrence of, *Hymeniacidon perlevis* were mostly similar among the models considered. For the remaining sets of predictors the partial effect had similar form among some models and differed for the others.

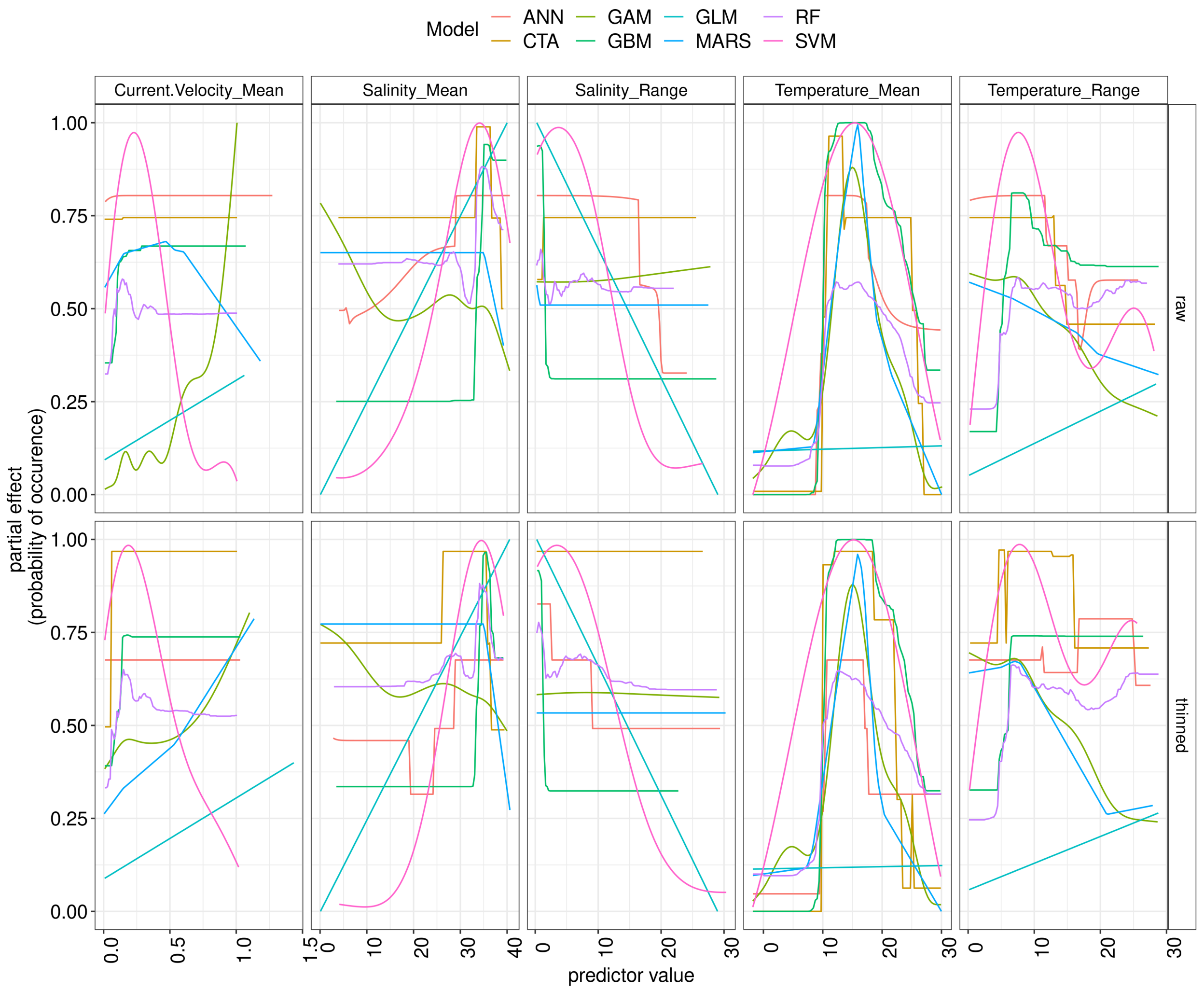

Figure 5: Partial effects of the predictors in influencing the distributon of *Hymeniacidon perlevis* in the eight models. Shown for the thinned and raw data

Predicted distribution of maps, the probability of occurrence, of *Hymeniacidon perlevis* from each of the eight models and the ensemble are shown in Figure 6.

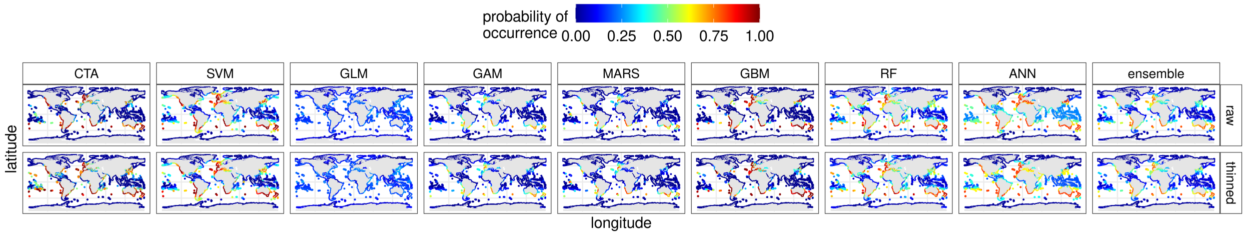

Figure 6: Predicted distribution of *Hymeniacidon perlevis*, based on each of the eight models, and the ensemble model. Shown for the thinned and raw data.

Figure 7 specifically shows the ensemble prediction on the raw and thinned data. They both show high probability of occurrence of *Hymeniacidon perlevis* on around the coast of the UK, off the coast of South Africa, off Argentina in South America, South coast of NewZealand, west coast of USA,…etc. Visually the predicted probability of occurrence were not that different between models based on the thinned or raw occurrence data.

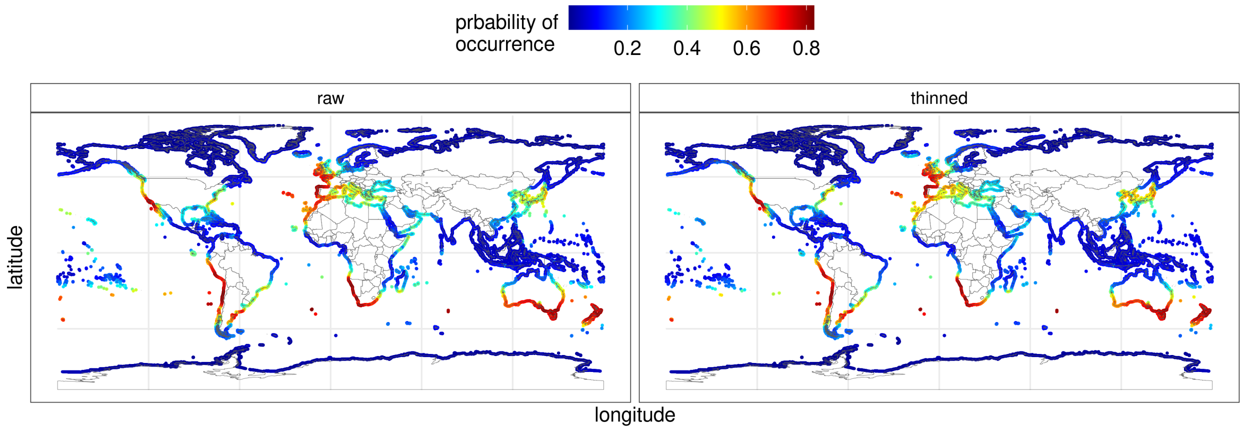

Figure 7: Predicted distribution of *Hymeniacidon perlevis* based on the ensemble model. Shown for the thinned and raw data.

Prediction uncertainty for the raw and thinned data are shown in Figure 8. The prediction uncertainty do appear to vary between the raw and thinned occurrence data and it was generally higher for the raw occurrence data. Regionally for example the uncertainty off the coast of South Africa appear to be relatively high for the raw data compared to the thinned data.

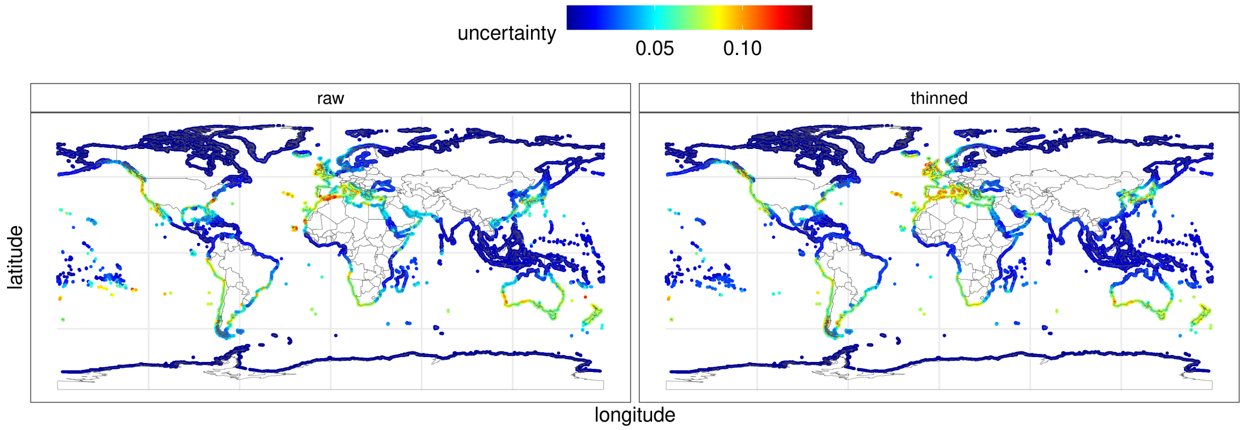

Figure 8: Prediction uncertainty for the thinned and raw data.

5 Discussion

[To be added]

6 Appenedix

Layers of environmental variables in modelling the distribution of *H. perlevis* and occurrence data with the randomly generated background data (pseudo-absence) will be in Appendices A and B respectively.

6.1 Appendix A

The environmental layers for the five variables: mean surface temperature, range of surface temperature, mean surface salinity, range of surface salinity and mean surface current velocity are presented below.

The global mean surface temperature for the coastal strip (within 10km of the coastline) are shown in Figure 9, similarly the data for range of surface temperature are shown in Figure 10, the corresponding data for the mean and range of surface salinity are shown in Figure 11 and Figure 12. The data for surfac current velocity for the coastal strip is shown in Figure 13

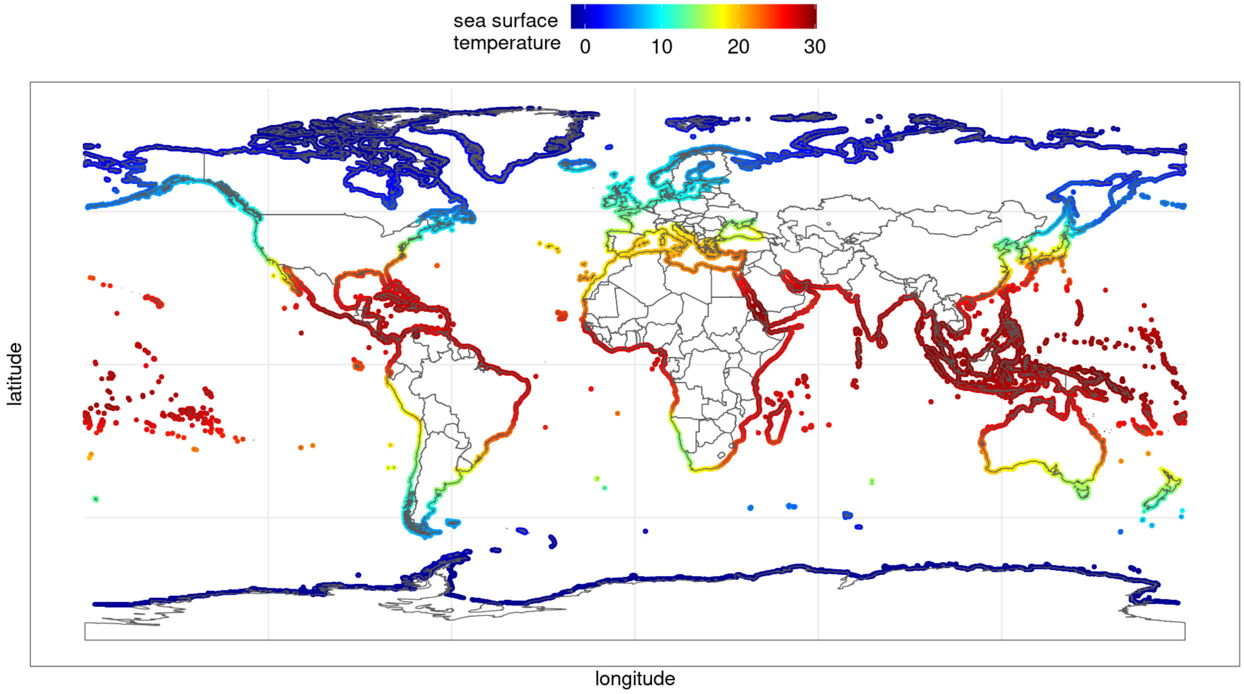

Figure 9: Global mean surface temperature for coastal strip (within 10km of the coastline).

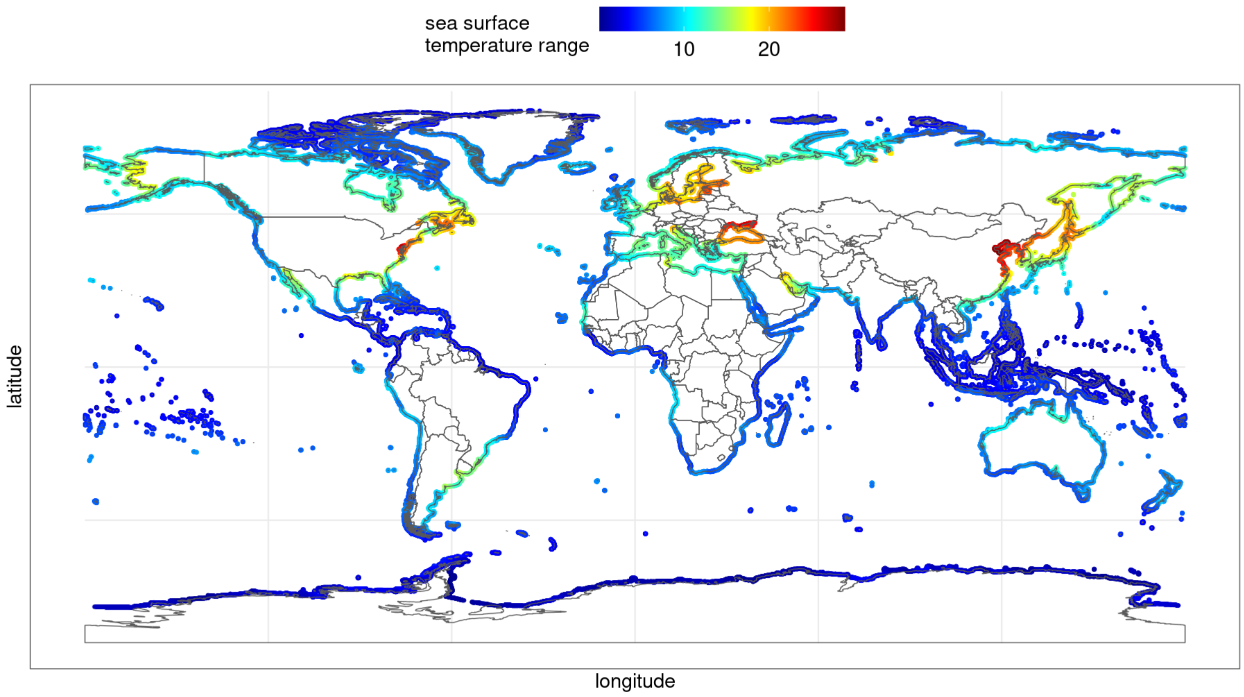

Figure 10: Global range for surface temperature for coastal strip (within 10km of the coastline).

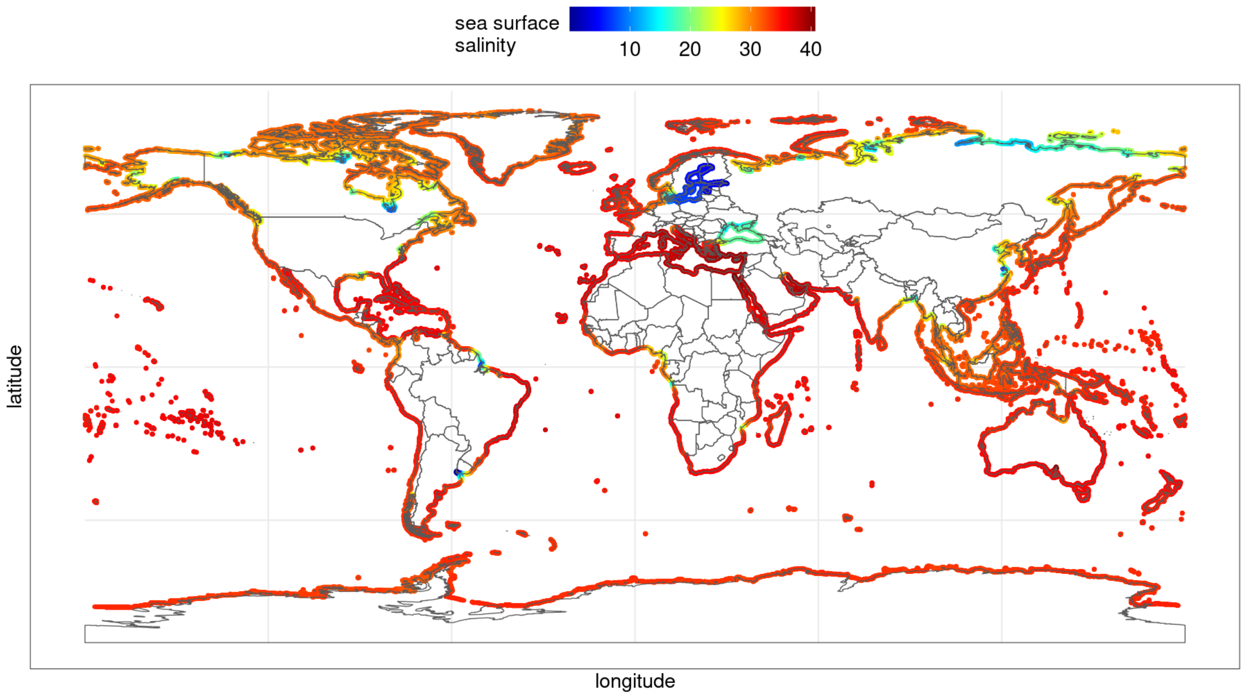

Figure 11: Global mean surface salinity for coastal strip (within 10km of the coastline).

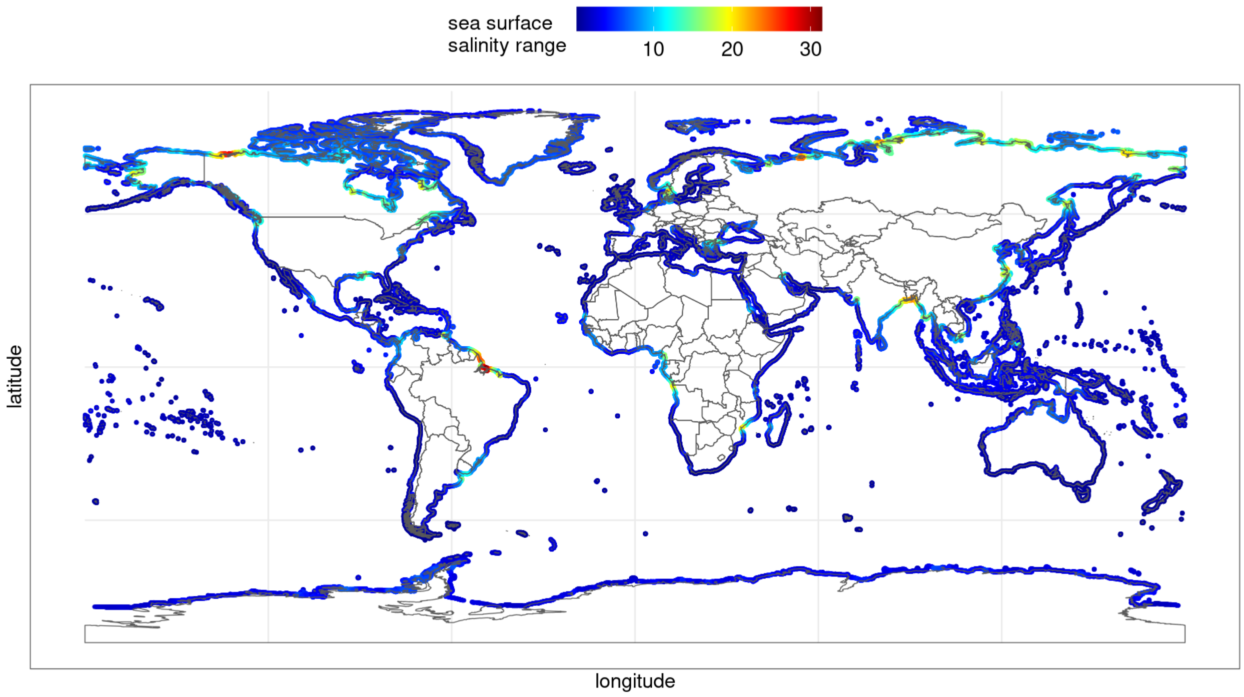

Figure 12: Global range for surface salinity for coastal strip (within 10km of the coastline).

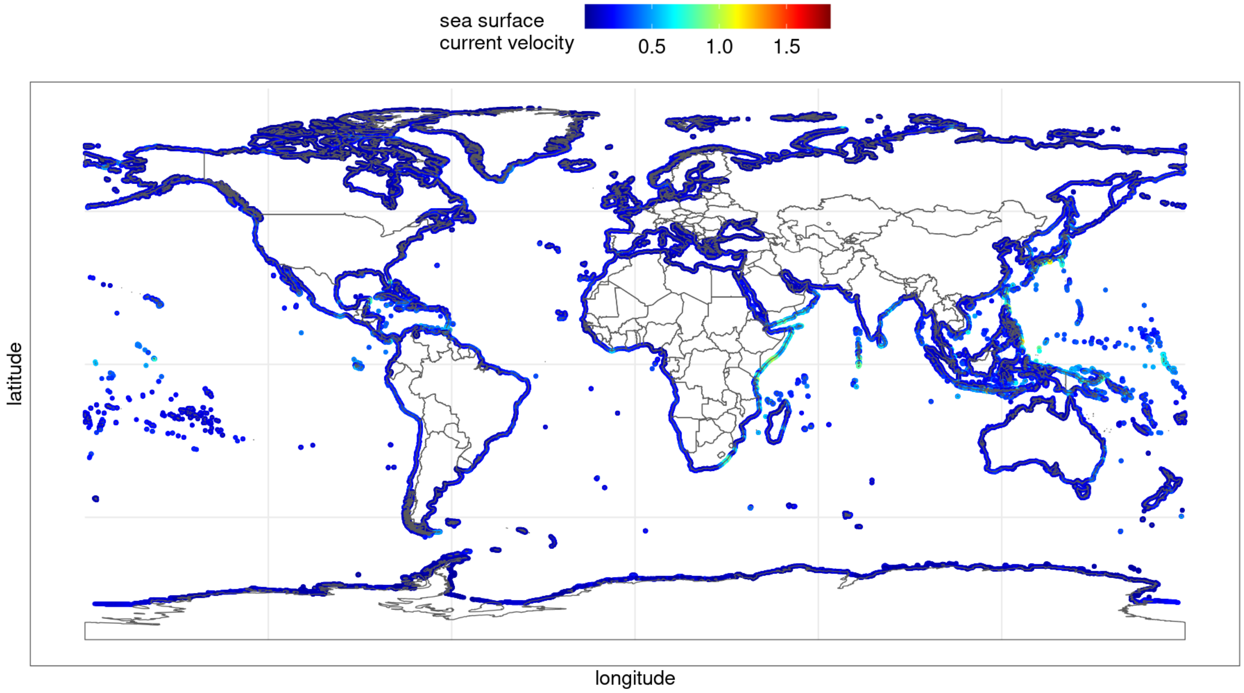

Figure 13: Global mean surface current velocity for coastal strip (within 10km of the coastline).

6.2 Appendix B

The randomly generated background (pseudo-absence) locations and occurrences/encounter locations collated from multiple sources are shown below for each of the species distribution models considered in this study. To save space only the unthinned data used in the modelling are presented. The randomly generated background and encounter locations used for the eight models are shown below Figure 14 for CTA, Figure 15 for SVM, Figure 16 for GLM, Figure 17 for GAM, Figure 18 for MARS, Figure 19 for GBM, Figure 20 for RF and Figure 21 for ANN.

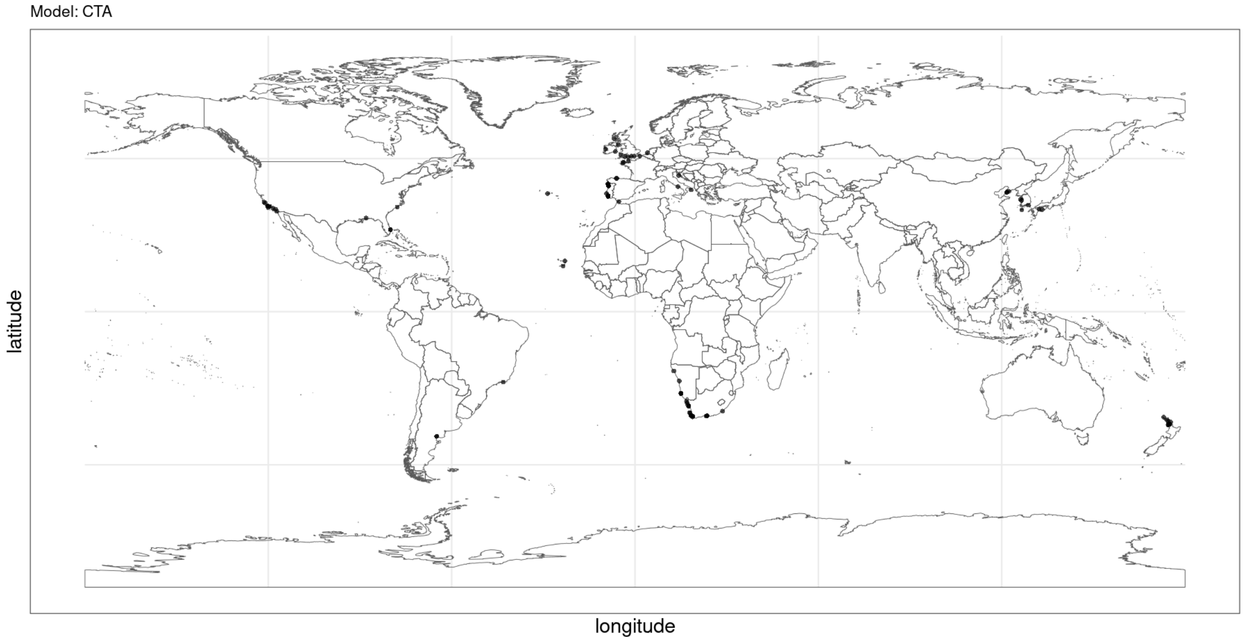

Figure 14: Global distribution of collatated occurrence/encounter (closed circle) locations for the unthinned data: CTA.

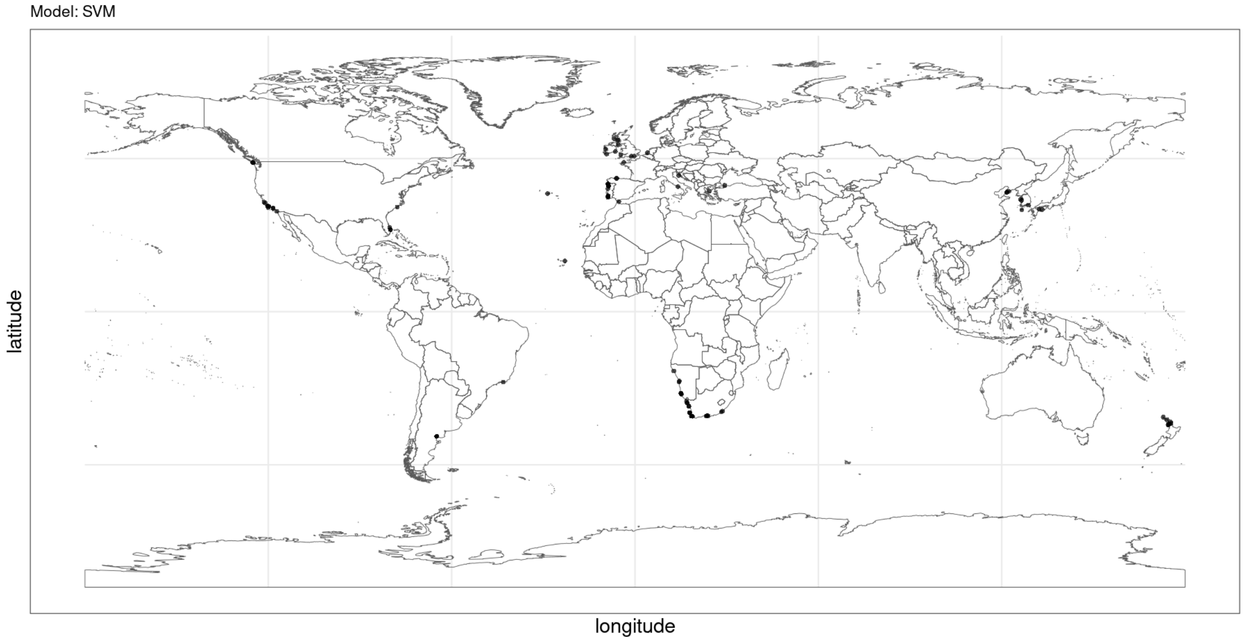

Figure 15: Global distribution of collatated occurrence/encounter (closed circle) locations for the unthinned data: SVM.

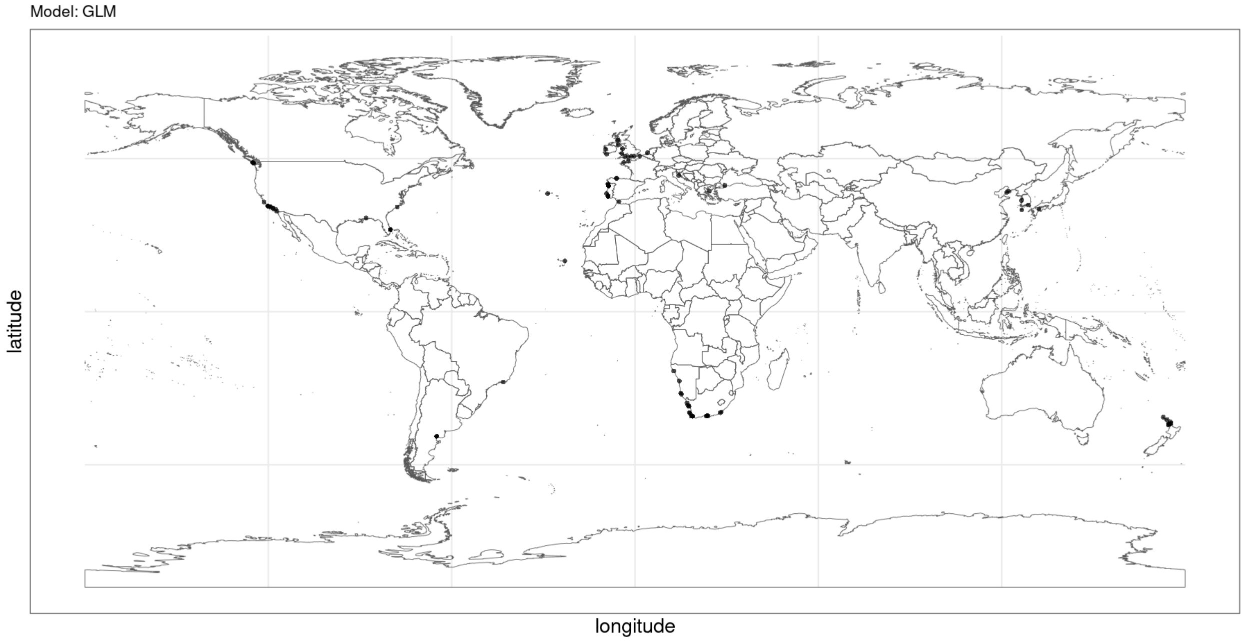

Figure 16: Global distribution of collatated occurrence/encounter (closed circle) locations for the unthinned data: GLM.

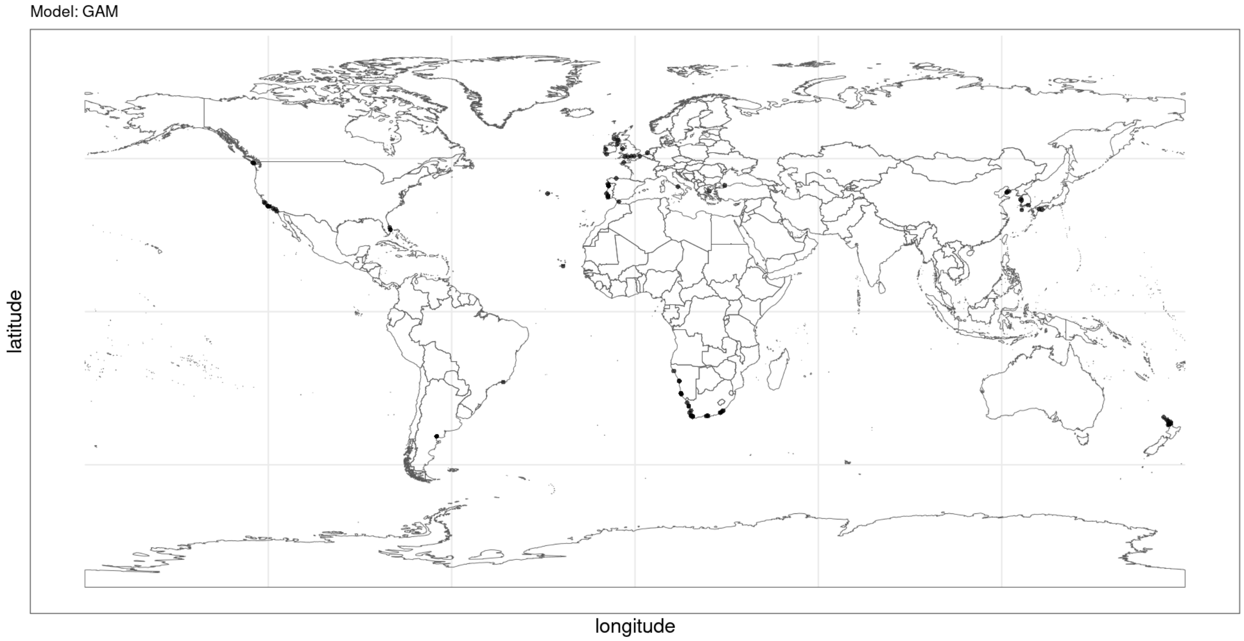

Figure 17: Global distribution of collatated occurrence/encounter (closed circle) locations for the unthinned data: GAM.

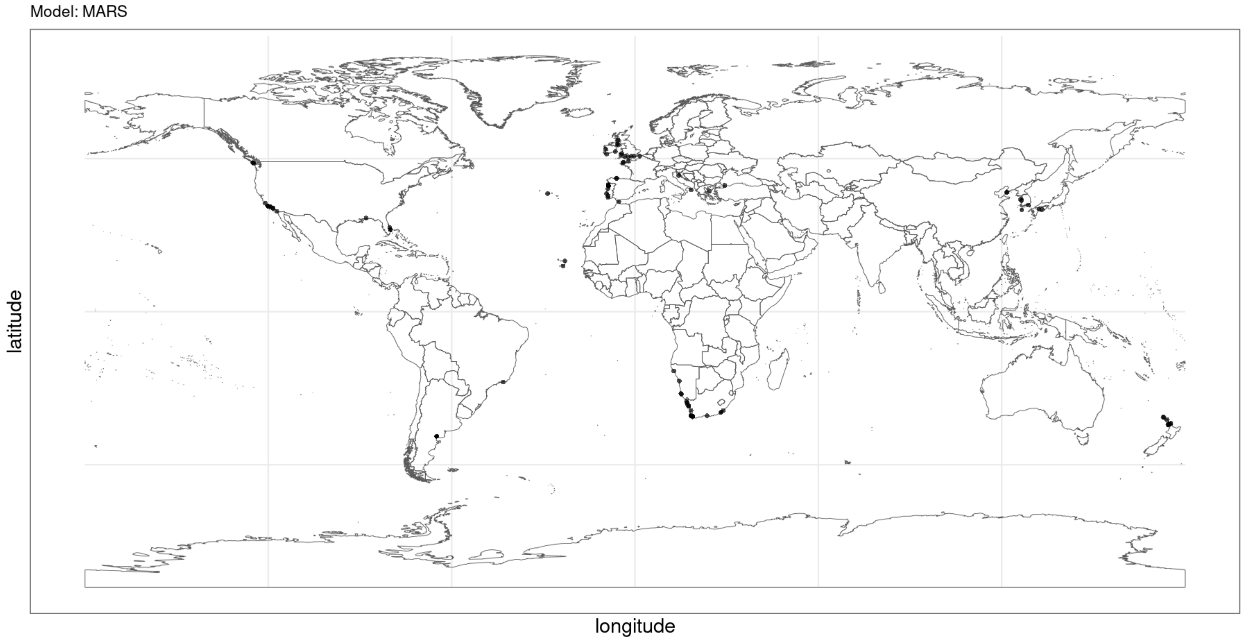

Figure 18: Global distribution of collatated occurrence/encounter (closed circle) locations for the unthinned data: MARS.

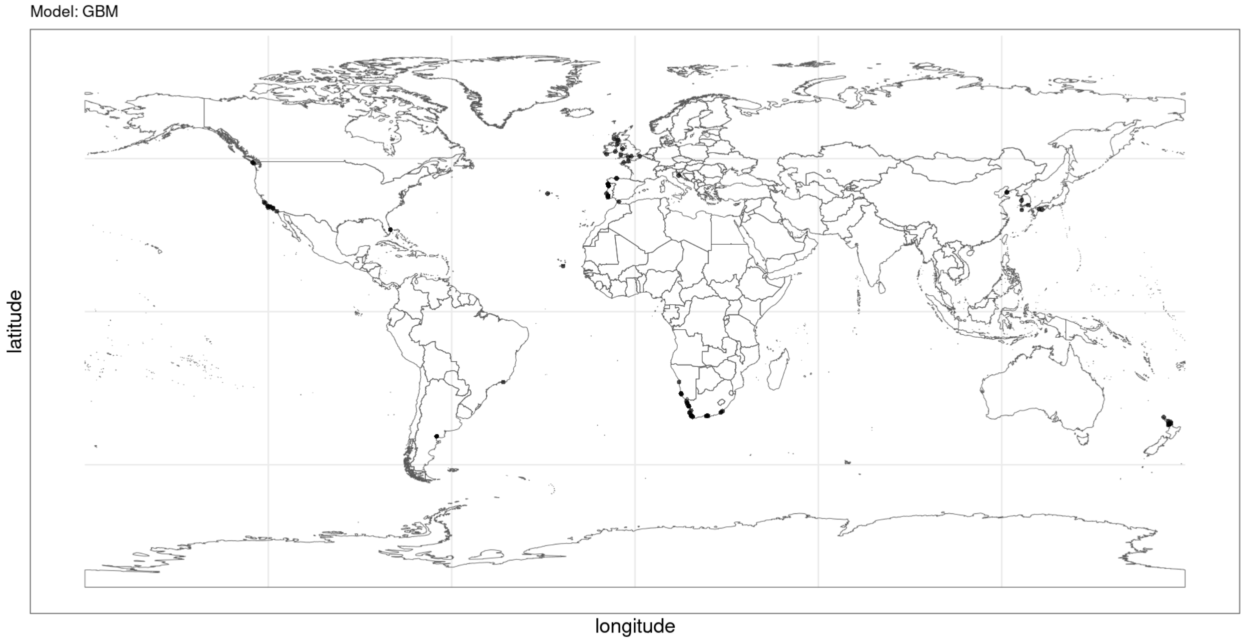

Figure 19: Global distribution of collatated occurrence/encounter (closed circle) locations for the unthinned data: GBM.

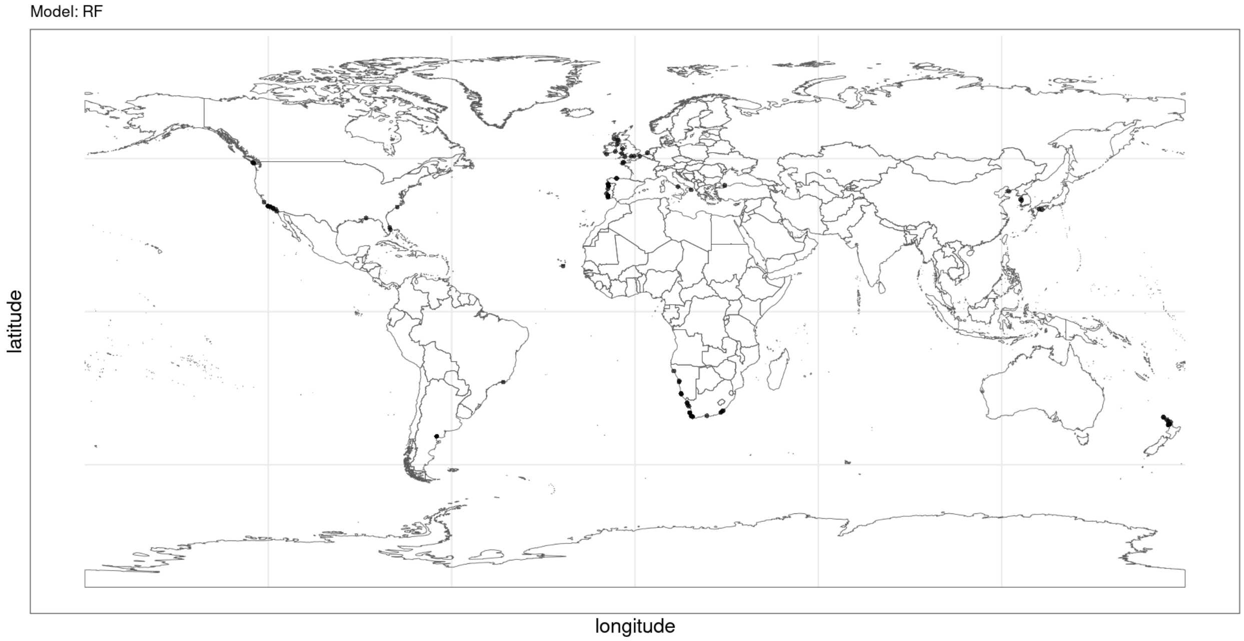

Figure 20: Global distribution of collatated occurrence/encounter (closed circle) locations for the unthinned data: RF.

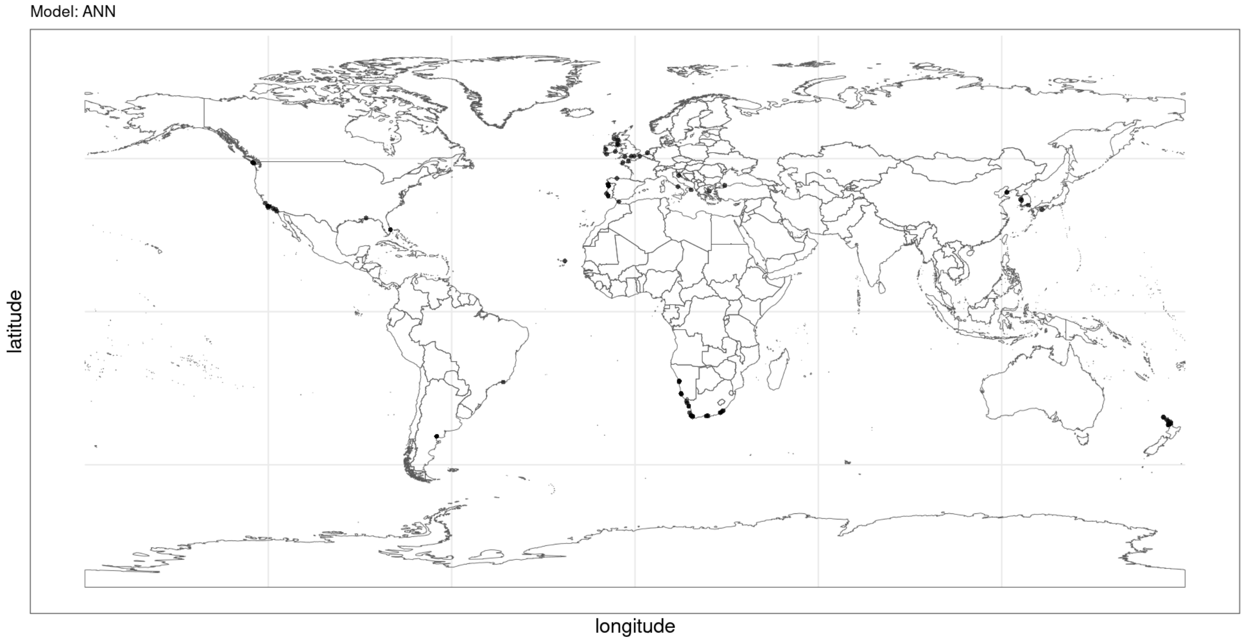

Figure 21: Global distribution of collatated occurrence/encounter (closed circle) locations for the unthinned data: ANN.

7 Session information

Table 1: System and session info for reproducibility

Setting	Value
version	R version 4.2.0 (2022-04-22)
os	Ubuntu 20.04.4 LTS
system	x86_64, linux-gnu
ui	X11
language	en_ZA
collate	en_ZA.UTF-8
ctype	en_ZA.UTF-8
tz	Africa/Johannesburg
date	2022-06-01
pandoc	2.17.1.1 @ /usr/lib/rstudio/bin/quarto/bin/ (via rmarkdown)

Table 2: R packages for reproducibility

	Package	Loaded version	Date
1	broom	0.8.0	2022-04-13
2	captioner	2.2.3	2015-07-16
3	devtools	2.4.3	2021-11-30
4	dplyr	1.0.9	2022-04-28
5	flextable	0.7.0	2022-03-06
6	forcats	0.5.1	2021-01-27
7	ggplot2	3.3.6	2022-05-03
8	ggrepel	0.9.1	2021-01-15
9	ggridges	0.5.3	2021-01-08
10	ggsn	0.5.0	2019-02-18
11	kableExtra	1.3.4	2021-02-20

	Package	Loaded version	Date
12	knitr	1.39	2022-04-26
13	magrittr	2.0.3	2022-03-30
14	purrr	0.3.4	2020-04-17
15	readr	2.1.2	2022-01-30
16	sf	1.0-8	2022-05-25
17	stringr	1.4.0	2019-02-10
18	tibble	3.1.7	2022-05-03
19	tidyr	1.2.0	2022-02-01
20	tidyverse	1.3.1	2021-04-15
21	usethis	2.1.5	2021-12-09
22	xtable	1.8-4	2019-04-21

References

Alathea, L. 2015. Captioner: Numbers figures and creates simple captions. https://github.com/adletaw/captioner.

Allaire, J., Xie, Y., McPherson, J., Luraschi, J., Ushey, K., Atkins, A., Wickham, H., *et al.* 2022. Rmarkdown: Dynamic documents for r.

Barbet-Massin, M., Jiguet, F., Albert, C. H., and Thuiller, W. 2012. Selecting pseudo-absences for species distribution models: How, where and how many? Methods in ecology and evolution, 3: 327–338. Wiley Online Library.

Henry, L., and Wickham, H. 2020. Purrr: Functional programming tools. https://CRAN.R-project.org/package=purrr.

R Core Team. 2022. R: A language and environment for statistical computing. R Foundation for Statistical Computing, Vienna, Austria. https://www.R-project.org/.

Robinson, D., Hayes, A., and Couch, S. 2022. Broom: Convert statistical objects into tidy tibbles. https://CRAN.R-project.org/package=broom.

Thuiller, W., Lafourcade, B., Engler, R., and Araújo, M. B. 2009. BIOMOD–a platform for ensemble forecasting of species distributions. Ecography, 32: 369–373. Wiley Online Library.

Turner, T. 2020. The marine sponge hymeniacidon perlevis is a globally-distributed exotic species. Aquatic Invasions, 15.

Wickham, H., Chang, W., Henry, L., Pedersen, T. L., Takahashi, K., Wilke, C., Woo, K., *et al.* 2022a. ggplot2: Create elegant data visualisations using the grammar of graphics. https://CRAN.R-project.org/package=ggplot2.

Wickham, H., François, R., Henry, L., and Müller, K. 2022b. Dplyr: A grammar of data manipulation. https://CRAN.R-project.org/package=dplyr.

Xie, Y. 2022. Knitr: A general-purpose package for dynamic report generation in r. https://yihui.org/knitr/.
