# Supplementary material for: Confirmation of the southern African distribution of the marine sponge Hymeniacidon perlevis (Montagu, 1814) in the context of its global dispersal
Source: PeerJ. 2022 Nov 25;10:e14388. doi: 10.7717/peerj.14388 (PMC9703993; doi:10.7717/peerj.14388)
Supplement: Figure S1 [file peerj-10-14388-s007.pdf]

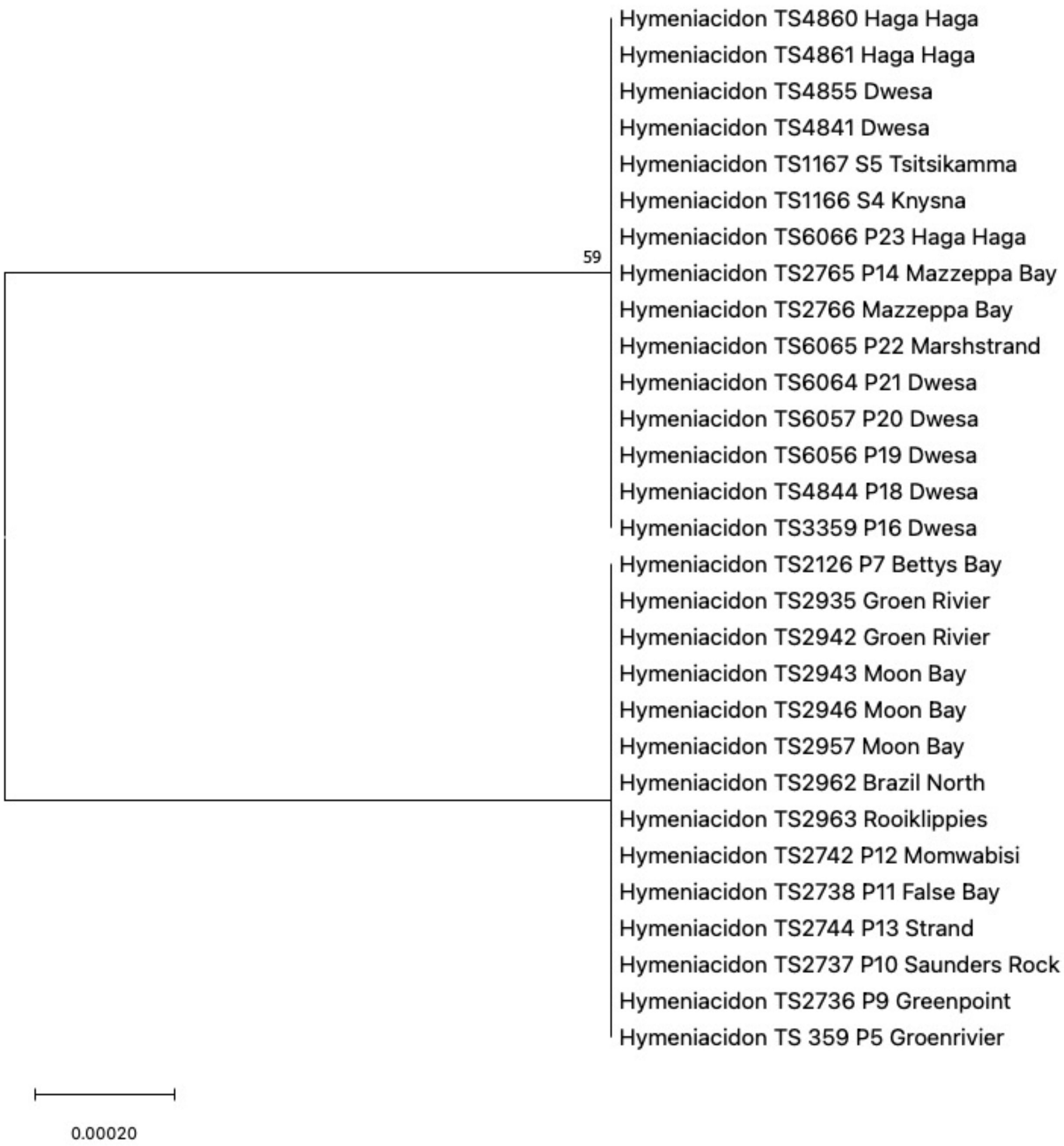

Hymeniacidon TS4860 Haga Haga
Hymeniacidon TS4861 Haga Haga
Hymeniacidon TS4855 Dwesa
Hymeniacidon TS4841 Dwesa
Hymeniacidon TS1167 S5 Tsitsikamma
Hymeniacidon TS1166 S4 Knysna
Hymeniacidon TS6066 P23 Haga Haga
59
Hymeniacidon TS2765 P14 Mazzeppa Bay
Hymeniacidon TS2766 Mazzeppa Bay
Hymeniacidon TS6065 P22 Marshstrand
Hymeniacidon TS6064 P21 Dwesa
Hymeniacidon TS6057 P20 Dwesa
Hymeniacidon TS6056 P19 Dwesa
Hymeniacidon TS4844 P18 Dwesa
Hymeniacidon TS3359 P16 Dwesa
Hymeniacidon TS2126 P7 Bettys Bay
Hymeniacidon TS2935 Groen Rivier
Hymeniacidon TS2942 Groen Rivier
Hymeniacidon TS2943 Moon Bay
Hymeniacidon TS2946 Moon Bay
Hymeniacidon TS2957 Moon Bay
Hymeniacidon TS2962 Brazil North
Hymeniacidon TS2963 Rooiklippies
Hymeniacidon TS2742 P12 Momwabisi
Hymeniacidon TS2738 P11 False Bay
Hymeniacidon TS2744 P13 Strand
Hymeniacidon TS2737 P10 Saunders Rock
Hymeniacidon TS2736 P9 Greenpoint
Hymeniacidon TS 359 P5 Groenrivier
0.00020
