# Supplementary figures and images for: Confirmation of the southern African distribution of the marine sponge Hymeniacidon perlevis (Montagu, 1814) in the context of its global dispersal
Source: PeerJ. 2022 Nov 25;10:e14388. doi: 10.7717/peerj.14388 (PMC9703993; doi:10.7717/peerj.14388)

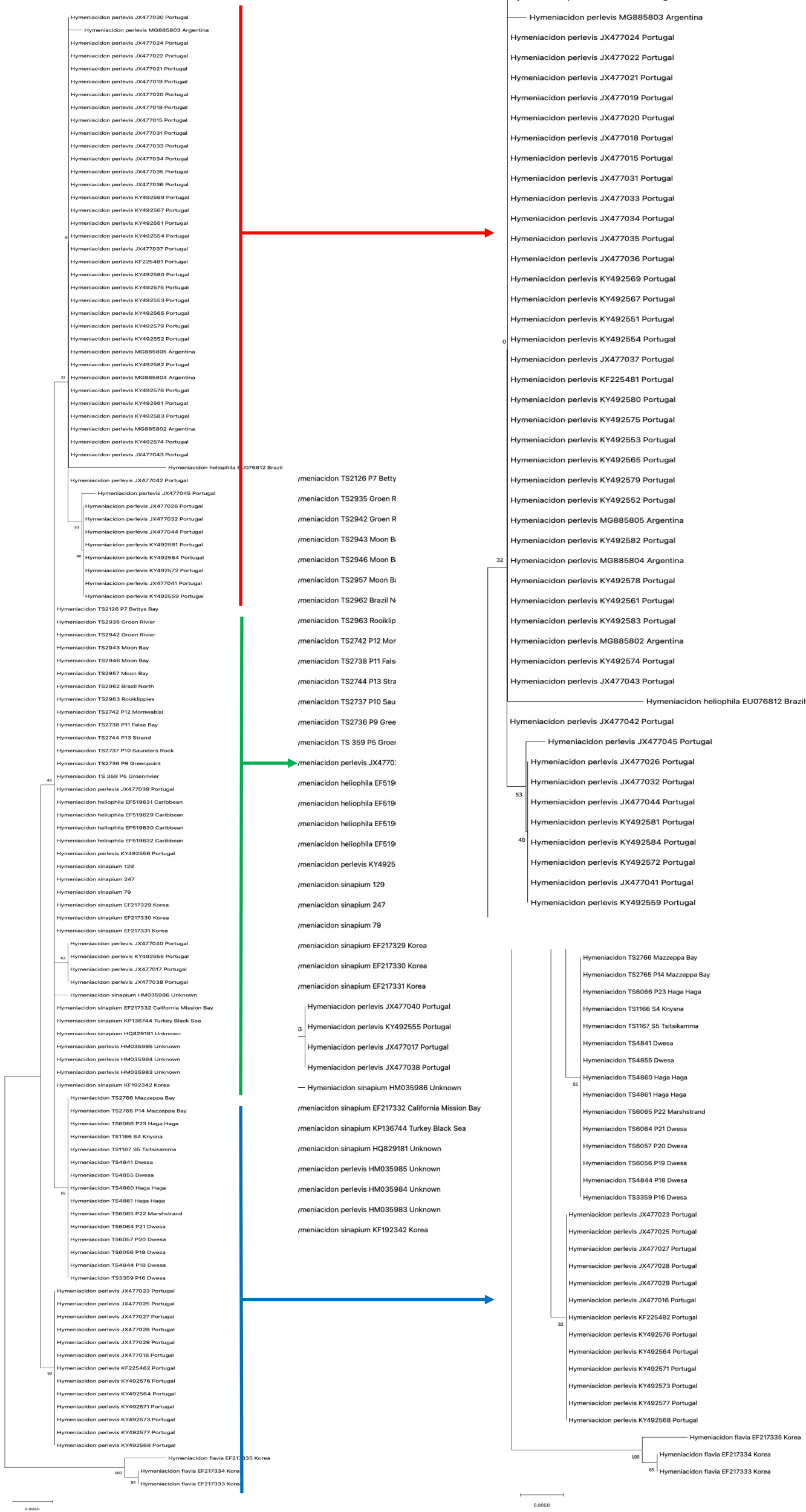

Supplement: Figure S2 — Numbers at nodes are bootstrap support values. Sequences are listed by their accession numbers, followed by taxon name and locality. [file peerj-10-14388-s008.pdf]
